# Medical Society of the County of Albany

**Published:** 1879-01

**Authors:** T. K. Perry

**Affiliations:** Secretary


*ART. II.—Medical Society of the County of Albany.*—Semi-Monthly Meeting, Nov. 6th, 1878. Reported by T. K. PERRY, M. D., Secretary.

The Society met at the usual hour and place, at the city hall, in Albany, to hold the first of its semi-monthly meetings for the season, the custom being to meet twice a month during the interval between the annual meeting in October, and the semi-annual in May.

The President, Dr. F. C. Curtis, called the meeting to order, and read an inaugural address. After referring to the fact that the work of the seventy-third year of the Society commenced with the meeting, and expressing thanks for the honor of being elected to the office, and the hope that "the meetings might be a gathering place for all the experience which every day brings to individuals," he made a number of propositions, some of which being of general interest, are as follows:

Why should not this Society be an influential power in the community? I think no one can tell me why it should not; I can easily see why it should. It is a trite saying that the medical profession is the conservator of the public health, and any one who gives a moment's thought to the history of its work in the past and sees the lives saved by vaccination, and the developed subject of hygiene in all its directions, will at once say this is true. The knowledge as to how to prevent disease, as far as it has reached, lies, equally with the knowledge how to cure it, with our profession. I do not know that any member of our guild has ever raised the question whether prevention of disease would not diminish the amount of work for which we are paid—the curing of it. It is required of a liberal profession that it shonld be liberal, and the demand cannot be stronger on any than it is on ours, since the power for good is greater. For the whole matter, as a whole, of dealing with disease—its history, its causation, its mode of development, under what circumstances it develops most, and how it is to be reduced to a minimum—its prevention and its cure—lies altogether in the hands of medical men. It is a subject lying close to the surface in the minds of all men, it is the great bane of humanity, and yet none have a practical knowledge of it excepting us. It is our study and work to make our fellow-men as free from it as possible.

Handling then, as a familiar subject, this one of disease, a subject that all allow to be so intensely important, does not the way appear clear how a medical society can, as a society, make itself highly useful to the community in which it is centered, and, in so far as it is useful, influential? Have I not hinted what should be a laudable ambition of this society? But has it heretofore lived up to what should be its duty to this community, and has it attained that point of influence which might be its due by an inherent right? I think not. To my knowledge, no question of hygiene has ever been submitted to its discussion but once, when the common council asked its opinion in regard to the use of the river water for a city water supply, and then its opinion was neither waited for or treated with any respect. There was a question which naturally fell to us upon which to give a weighty and respected opinion, but we had not then, nor since have we, as a society, won the right to consideration even by action in such matters.

My first proposition then is, that this society owes a duty to this community, at the point where it touches it, in regard to hygiene and the conservation of the public health, and that by attention to this it will give itself a weight, and secure attention to itself from its surroundings, that will accrue to it in no other way. To make a start in effecting this, I submit that, first, a committee of influential members who have made the subject more or less a study, be appointed from year to year, a standing committee on hygiene, and the relation of the profession to the public; that this committee put itself in communication with the health authorities of the city and county, and with the State Society Committee on Hygiene; that they make a special study of the topography of the county, of the drainage of the cities, of the condition of the houses here as to drainage, dampness, etc.; that they keep close note of prevailing diseases, especially zymotic, the locality and center of such as may be endemic or epidemic; that they look after the condition of the schools as regards the care for the health of the scholars, and the amount of transmission of disease to them from infected places, (a point we frequently have in question,) and in various ways, by the authority of this society, to press itself forward to investigate the lack of healthfulness of localities, and suggest remedies. Is it not apparent that such a committee, filled with a personal interest, desirous of the public good, and anxious to facilitate the best interests of our calling, might accomplish very much, and little by little push into public notice the benefit of the society to the people, gradually winning for it a place of earnest appreciation and respect in the public mind. It may even be that at some time, instead of a Health Board of Aldermen, we may have in Albany, one of physicians, with all the autocratic powers the law gives to it. But no committee could do this work single-handed; it must have the co-operation of the entire society and the assistance of every individual. Members should report to it all cases of zymotic disease, all suspected houses, all stagnant pools, schools that are admitting infected children, and, if necessary, ask their assistance to correct prevailing evils. In making these suggestions regarding the duty of this society to this community, I do not wish to ignore the fact that we have already a committee on hygiene, which we do have in pursuance of the wish of the State Medical Society, but to propose making it more useful to ourselves, and in the various relations of the profession, to the public.

The following extract from a letter by Dr. W. C. Wey, of Elmira, whose sanitary work in the schools of his city is well known, in reply to an inquiry concerning the part taken in public hygiene by the Medical Society of Chemung County, will greatly add to the weight of my own words. He says :

"The initiative in all matters of school hygiene and general hygiene, as it applies to individuals, families, trades, municipal affairs, such as the construction of private and public buildings, sewage, the filling up of low lands, interference with natural water-courses,

&c., &c., should come from the medical profession, through the agency, and by the authority of the various organizations which constitute guilds, as it were, for concerted action in modifying popular sentiment, and in hastening processes of reform. In regard to Boards of Education, it is highly important that medical men should be represented in them. An extensive field of inquiry and improvement in matters affecting not only the health of children, but the efficiency of schools is thus open to medical scrutiny, and the correction of errors is within the power of persistent professional endeavor. I can only mention to condemn the modifications of municipal law by which aldermen in cities, sitting in council, are clothed with authority as Boards of Health. The impotency of the Common Council, acting as a Board of Health, has been illustrated during the course of an epidemic of diphtheria in this city, at this time in full force. which prevailed for more than a year before official action was taken in regard to it, and such action as has been taken is unwise, unsanitary, halting and half-hearted. * * * * I hope that your society will adopt measures in connection with this subject which will advance the intesests of sanitary science, not only in the city and county of Albany, but in a more comprehensive manner, looking towards the creation of a State Board of Health, about which much might be said."

I have a second proposition to make—one that relates to our own private advantages. It is that we have another standing committee—a committee on Pathology. This may well be a large committee, sufficiently so to compose a respectable meeting of itself, to discuss material presented to it directly, or through the society. It should be made up of those specially interested in this study. As they may see fit, reports might be presented of the results of their investigations to the society.

Now I know that it has been argued that a County Medical Society cannot, from its nature and composition, be made a scientific success. It is said that, being an incorporated society, almost necessarily including in its membership all physicians in the county limits, the State law, at one time, rendering this obligatory, and it being still considered a point of honor for all regular physicians to join their county societies, its function is principally to gather in a body, the reputable medical men in a civil or business way, to put on record by membership in it that a man is a regular physician, to carry out the laws as imposed upon it, and from the very fact that it contains all,that its elements are too diverse,and contain too great a lack of sympathy of mind to make it possible for it to devote itself to study and scientific advancement. And, really there may be truth in this, and there is great need that present feeling, unkindly criticism, discussive talk born of too little reflection on the subjects announced, and all jarring elements should be held in check. But if we come together, all of us, with the desire to learn something, and with study of the subjects to contribute something, I can see no reason why the county society should not fill all the indications aimed at by any form of medical society. I think this may be made more easily possible, by having such a committee as I have indicated, for the more careful study of the abstruser subjects that may come before the society.

The address contained other propositions of a more local character—that a nominating committee be elected ; also a committee on registration, whose duty it should be to carry out the suggestions of the State Society, and also have charge of the society Chronological list, and print the Annual Register; calling attention to a by-law of the State Society requiring the Board of Censors to examine into the educational qualifications of young men proposing to commence the study of medicine; and also to another resolution requiring that five copies of the State Society Transactions be taken for each delegate sent from the county, and proposing that the annual dues be reduced to the legal sum of one dollar. In conclusion, the assistance of every member was solicited, to further the efforts of the executive officers to make the year a profitable one in every way to the society.

On motion, a committee on the President's Address was appointed, which subsequently reported, favoring the various propositions submitted.

A committee appointed at a former meeting to examine into the status of members not residing in the county limits, under the code of by-laws recently adopted, made a report. The article bearing on the question is as follows: "Any member, not in arrears with the society, who may permanently remove from the county of Albany, shall be known as a non-resident member. Such members shall not be required to contribute to the funds of the society; shall not vote at any election, be eligible to any office, or receive notices or publications from or through the society, but, in all other respects, shall enjoy every right and privilege of a resident member. Physicians residing out of the county may be elected to non-resident membership."

After reviewing the history of the matter, which led to the incorporation of this article in the by-laws, and giving the action of the State Society upon it, the committee came to the following conclusions: That, under the by-laws, no one not a resident of the county could be elected to full membership; that any one who at any time in the past had been elected a member while residing in the county, and subsequently removed from it should be classed as a "non-resident" member; and that those who had been elected members while residing out of the county could not be construed by this by-law to be "non-resident" members, but must be considered as full members, and their status not affected. It was proposed that it be put to their option whether they continue as such, or resign to "non-resident" membership. Of these there were seven, living just over the river in Rensselaer County. The report was adopted.

Dr. S. B. Ward then read a paper entitled "A case involving difficulties in determining the possibility of conception, and subsequent diagnosis of pregnancy."

This is to appear in a forthcoming number of the Obstetrical Journal.

The points in this paper which were especially significant from their tendency to baffle diagnosis, were:

1. The facts admitted by both parties, that there had never been connection, the nearest approach thereto having been contact of the parts merely, the emission taking place entirely outside the vulva.

2. The very natural and reasonable supposition that the non-appearance of the menses in December was due to the severe cold and general illness of the patient at the time.

3. The severe pains experienced at the January epoch so characteristic of uterine contraction, were no more severe, and differed in no respect from those occurring two years previous, when the patient's virginity was beyond question.

4. The occurrence during this time of a severe and protracted attack of Intermittent Fever, accompanied by an unusually high range of temperature, tended to embarrass and render still more obscure all efforts at diagnosis.

DISCUSSION.

Dr. J. S. Mosher thought the case in many respects very unusual. Recalled a case in his own practice, of an unmarried woman in whom pregnahcy existed, there never having been contact. Did not think the cessation of the menses any criterion to be guided by.

Dr. Van DerVeer spoke of a case where a young lady of seventeen became pregnant without ever having complete intercourse. also of one where a tramp had attempted an outrage, but had almost entirely failed in his purpose, owing to the victim's struggles. She, however, became pregnant, and was confined at term.

Dr. J. L. Babcock mentioned a case recorded in the Edinburgh Medical and Surgical Journal, of a man whose penis had been amputated close to the pubis, who, nevertheless succeeded in fecundating his wife.

Dr. E. R. Hun, reported a remarkable case of what he believed to have been protracted gestation. The date of expected confinement was April 23d. Labor did not, however, supervene until August 4th, of same year. Knows no other explanation of the discrepancy.

Dr. S. B. Ward thought Orulation and Menstruation in some instances to be separate and distinct. Concerning the longevity of Spermatozoa, thought it a settled point that they could exist under certain conditions for eight days at least.

Dr. F. C. Curtis, in speaking of fecundation, said he believed that the prime factor consisted in the individual motion of the spermatozoa.

Following this discussion, Dr. J. S. Mosher spoke with especia reference to the Metric System and its no doubt eventual adoption by all nations; and to further its more general use in our own community, as well also to more thoroughly familiarize ourselves as professional men, with its advantages: Moved—"That a committee be appointed to consider and report at a subsequent meeting, the best method whereby such a change could be secured.—Carried.

COMMITTEE.

Drs. J. S. Mosher, E. R. Hun, J. L. Babcock.

There being no further business before the society it was declared adjourned.

